# The impact of essential fatty acid ratios and unsaturated to saturated fat ratio on growth performance of grow-finish pigs and estrus detection of gilts

**DOI:** 10.1093/tas/txad088

**Published:** 2023-07-28

**Authors:** Spenser L Becker, Laura L Greiner

**Affiliations:** Department of Animal Science, Iowa State University, Ames, IA 50011, USA; Department of Animal Science, Iowa State University, Ames, IA 50011, USA

**Keywords:** linoleic acid, linolenic acid, flaxseed oil, swine, lipids

## Abstract

The objective of this study was to investigate the effects of dietary unsaturated and saturated fat ratio (U:S) and the ratio of linoleic and linolenic acid (LA:ALA) on the growth performance of grow-finish pigs and estrus detection of gilts. A total of 240 pigs with initial body weight (BW) 54.4 ± 5.5 kg were randomly assigned to a high (>1.8; HUS) or low (<1.0; LUS) U:S in combination with a high (20:1), moderate (12:1), or low (4:1) LA:ALA in a 3 × 2 factorial arrangement. Dietary ratios were achieved using blends of choice white grease, beef tallow, corn oil, flaxseed oil, or palm kernel oil. Diets were fed across three phases and balanced for energy and LA. Pigs were housed across 60 pens with either four gilts or four barrows per pen. On day 49, 1 gilt per pen was moved to individual housing at approximately 154 d of age for evaluation of reproductive characteristics. Data were analyzed as repeated measures using PROC MIXED (SAS 9.4; SAS Inst., Cary, NC) with pen as the experimental unit and U:S, LA:ALA, sex, and their interactions as fixed effects. Initial BW was fit as a covariate. Within each phase, there were no differences in BW, daily gain (ADG), feed intake (ADFI), or feed efficiency (G:F) for U:S, LA:ALA, or their interaction when averaged across sex (*P* ≥0.128). Gilt feed efficiency was improved during the second phase compared to barrows; however, feed efficiency was not different between barrows and gilts during the first and third phases; resulting in a similar feed efficiency between sexes for the overall period (*P* = 0.523). Compared to HUS, gilts receiving LUS had higher ADFI overall (*P = *0.018), which translated into improved G:F for HUS gilts (*P = *0.011). Overall, gilts receiving the 20:1 diet tended to have improved G:F compared to 12:1 (*P *= 0.086). ADG was improved in pigs fed diets formulated with unsaturated fat sources to a 20:1 LA:ALA, regardless of sex. Detection of first estrus by 235 d of age in gilts was not impacted by U:S or LA:ALA (*P ≥* 0.356). In conclusion, feeding differing dietary U:S and LA:ALA ratios impacts growth of growing pigs, particularly improving feed efficiency of gilts fed diets with unsaturated fat sources or a 20:1 LA:ALA. Further investigation into the physiological mechanisms differentially affecting gilt growth when fed varying dietary LA:ALA is warranted to understand the impact on reproductive outcomes.

## Introduction

Dietary lipids are highly digestible energy sources for pigs; however, the digestion and utilization of those lipids are highly dependent on the source and degree of saturation (Kerr et al., 2015). Generally, the digestibility of lipids increases with increasing dietary unsaturated fat to saturated fat ratio (U:S; Wiseman and Agunbiade, 1998). Current swine diets in the United States tend to have higher unsaturated fat content due to the high inclusion of corn and soybean meal, though they can vary in their U:S depending on the supplemented dietary fat source utilized. Inclusion of animal-based fats, such as choice white grease, results in a lower U:S due to their high content of saturated fatty acids (SFAs; NRC, 2012). Plant-based fat sources, such as soybean oil and corn oil, have a high LA (C18:2) content, resulting in high dietary U:S ratios (NRC, 2012).

Previous work in pigs has demonstrated that a dietary fat source high in unsaturated FAs can improve average daily gain (ADG; Morgan et al., 1992; Suomi et al., 1993); however, conflicting results have been shown feeding varying dietary fat sources to modern swine genetics (Liu et al., 2018). Furthermore, there has been a growing interest in optimizing the dietary ratio of linoleic acid to alpha-linolenic acid (LA:ALA). In other stages of pork production and across species, it has been shown that altering this ratio can improve growth performance and immune function (Wu and Chen, 2012; Tanghe et al., 2014; Che et al., 2019), though there is a paucity of data in growing pigs. Becker et al. (2023) reported an improvement in final body weight (BW) and ADG in gilts fed a LA:ALA of 12:1, regardless of dietary energy level. A secondary study showed an improvement in final gilt BW and ADG when fed isocaloric diets with a 20:1 LA:ALA, regardless of LA inclusion (Becker and Greiner, 2021). Both studies reported a beneficial alteration in gilt growth, though at differing LA:ALA ratios. The authors hypothesize this is potentially due to alterations in hormone production, as FAs serve as important precursors in their synthetic pathway. Additionally, diets in both studies had varying U:S ratios. Therefore, the objective of this study was to investigate the effects of dietary U:S and the fatty acid (FA) ratio of LA and ALA on the growth performance of grow-finish pigs and estrus detection of gilts.

## Materials and Methods

All experimental procedures adhered to guidelines for the ethical and humane use of animals for research according to the Guide for the Care and Use of Agricultural Animals in Research and Teaching (FASS, 2010) and were approved by the Iowa State University Institutional Animal Care and Use Committee (IACUC #21-047).

### Animals, Diets, and Experimental Design

A total of 240 growing pigs (PIC Genus 337 × 1050, Hendersonville, TN) were individually weighed and randomly allotted to pens such that there were either four barrows or four gilts per pen, for a total of 60 pens. Pigs had an initial BW of 54.4 ± 5.5 kg. Pens had partially slated, concrete flooring with dimensions of 1.83 m × 1.93 m. This study was conducted during the summer months in a central Iowa research farm. Pigs originated from a porcine reproductive and respiratory syndrome virus (PRRSv) vaccinated herd. One pig per pen was vaccinated for *Lawsonia intercellularis* (Porcilis ILEITIS; Merck Animal Health, Madison, New Jersey), porcine circovirus Type 2 and *Mycoplasma hyopneumoniae* (Circumvent PCV-M G2; Merck Animal Health) according to the manufacturer’s recommendations. Pigs did not display any clinical signs of disease and remained healthy throughout the 70-d trial.

Pens were randomly assigned to a high (>1.8; HUS) or low (<1.0; LUS) U:S with a high (20:1), moderate (12:1), or low (4:1) LA:ALA FA ratio in a 3 × 2 factorial design. The dietary LA:ALA ratios were selected based on previous research by Becker et al. (2023). Choice white grease, beef tallow, corn oil, and flaxseed oil were primarily utilized to formulate the dietary LA:ALA ratios. Palm kernel oil was used in the 4:1 LUS diet to maintain a U:S of less than 1.0. Diets were fed across one 14-d phase and two 28-d phases, for a total of three dietary phases. Diets were balanced for metabolizable energy and LA inclusion. Diets were presented in mash form and were primarily based on corn and soybean meal (Tables 1 to 3). A two-space galvanized steel feeder (width = 76 cm) with hinged lids and two nipple drinkers were used to provide ad libitum access to feed and water throughout the study. Analyzed FA content of the dietary fat sources and dietary treatments are presented in Tables 4 and 5, respectively. The diets were formulated to meet or exceed NRC (2012) nutrient recommendations of growing pigs and did not contain antibiotics or pharmaceutical levels of copper or zinc.

**Table 1.** Ingredient and nutrient composition of the grow-finish experimental diets, phase 1 (as-fed basis)

Item	U:S^1^ > 1.8			U:S < 1.0		
	20:1^2^	12:1	4:1	20:1	12:1	4:1
Ingredient, %						
Corn	72.74	72.78	72.55	72.58	72.58	72.37
Soybean meal	22.89	22.89	22.89	22.89	22.89	22.89
Beef tallow	–	–	–	0.41	0.45	–
Choice white grease	–	–	–	1.01	0.88	0.71
Corn oil	1.21	1.08	0.73	–	–	–
Flaxseed oil	0.06	0.18	0.73	0.03	0.11	0.57
Palm kernel oil	–	–	–	–	–	0.35
DL-methionine	0.10	0.10	0.10	0.10	0.10	0.10
L-lysine HCL	0.38	0.38	0.38	0.38	0.38	0.38
L-threonine	0.12	0.12	0.13	0.13	0.13	0.13
L-tryptophan	0.01	0.01	0.01	0.01	0.01	0.01
Premix^3^	0.35	0.35	0.35	0.35	0.35	0.35
Phytase^4^	0.02	0.02	0.02	0.02	0.02	0.02
Calcium carbonate	1.05	1.06	1.05	1.06	1.06	1.05
Monocalcium phosphate	0.56	0.54	0.56	0.54	0.54	0.56
Sodium chloride	0.50	0.50	0.50	0.50	0.50	0.50
Calculated nutrient levels						
SID Lys, %	1.03	1.03	1.04	1.03	1.03	1.04
SID Met: Lys	0.33	0.33	0.33	0.33	0.33	0.33
SID Thr: Lys	0.62	0.62	0.62	0.62	0.62	0.62
SID Trp:Lys	0.18	0.18	0.18	0.18	0.18	0.18
SID TSAA: Lys	0.57	0.57	0.57	0.57	0.57	0.57
Ca, %	0.70	0.70	0.70	0.70	0.70	0.70
STTD P, %	0.35	0.35	0.35	0.35	0.35	0.35
ME, Mcal/kg	3.34	3.33	3.34	3.34	3.34	3.34
SID Lys/ME, (g/kg)/Mcal	3.10	3.10	3.10	3.10	3.10	3.10
Analyzed nutrient levels						
Dry matter, %	88.64	88.12	88.28	88.30	88.46	88.49
GE, Mcal/kg	3.90	3.89	3.91	3.90	3.89	3.92
aEE, %	4.15	4.52	4.58	4.19	4.50	4.88

^1^Calculated dietary U:S - High = >1.8, Low = <1.0.

^2^Calculated FA ratio as dietary LA:ALA.

^3^Provided 6,614 IU vitamin A, 827 IU vitamin D, 26 IU vitamin E, 2.6 mg vitamin K, 29.8 mg niacin, 16.5 mg pantothenic acid, 5.0 mg riboflavin, and 0.023 mg vitamin B12, 165 mg Zn (zinc sulfate), 165 mg Fe (iron sulfate), 39 mg Mn (manganese sulfate), 17 mg Cu (copper sulfate), 0.3 mg I (calcium iodate), and 0.3 mg Se (sodium selenite) per kg of diet.

^4^450 FTU/kg of diet provided 0.06% Ca and 0.11% STTD P.

ME, metabolizable energy; STTD, standardized total tract digestible; SID, standardized ileal digestible; aEE, acid ether extract.

**Table 4.** FA composition of dietary fat sources

Common Name, %	FA	Tallow	Choice white grease	Flaxseed oil	Corn oil
Caprylic acid	8:0	0.00	0.01	0.00	0.00
Capric acid	10:0	0.04	0.09	0.00	0.00
Lauric acid	12:0	0.05	0.08	0.00	0.00
Myristic acid	14:0	2.79	1.34	0.04	0.03
Myristoleic acid	14:1	0.39	0.01	0.00	0.00
Pentadecanoic acid	15:0	0.42	0.05	0.01	0.00
Palmitic acid	16:0	24.02	23.98	5.52	11.43
Palmitoleic acid	16:1	1.90	2.31	0.07	0.08
Margaric acid	17:0	1.29	0.32	0.06	0.05
Heptadecenoic acid	17:1	0.59	0.23	0.03	0.02
Stearic acid	18:0	21.92	12.97	3.80	1.49
Oleic acid	18:1 c9	35.31	37.29	17.75	28.43
LA	18:2 n6	2.48	12.73	18.57	56.35
Arachidic acid	20:0	0.15	0.21	0.15	0.33
γ-ALA	18:3 n6	0.00	0.00	0.18	0.00
Eicosenoic acid	20:1	0.18	0.84	0.00	0.26
α-ALA	18:3 n3	0.23	0.46	53.03	0.96
Eicosadienoic acid	20:2 n6	0.06	0.69	0.05	0.05
Eicosatrienoic acid	20:3 n3	0.00	0.00	0.01	0.00
Dihomo-γ ALA	20:3 n6	0.00	0.00	0.02	0.11
Mead Acid	20:3 n9	0.00	0.02	0.02	0.00
Arachidonic acid	20:4 n6	0.05	0.29	0.00	0.01
Eicosapentaenoic acid	20:5 n3	0.04	0.04	0.02	0.01
Docosanoic acid	22:0	0.07	0.11	0.01	0.01
Docosenoic acid	22:1	0.00	0.07	0.05	0.00
Docosadienoic acid	22:2 n6	0.00	0.00	0.02	0.02
Docosapentaenoic acid	22:5 n3	0.00	0.05	0.02	0.00
Docosohexaenoic acid	22:6 n3	0.00	0.00	0.02	0.00

**Table 5.** FA composition of dietary treatments^1^

Analyzed FAs	U:S > 1.8			U:S < 1.0		
	20:1	12:1	4:1	20:1	12:1	4:1
Phase 1						
C12:0, %	–	–	–	–	–	0.14
C14:0, %	–	–	–	0.03	0.02	0.06
C18:0, %	0.07	0.07	0.09	0.27	0.27	0.26
C18:1, %	1.08	1.06	1.06	1.25	1.23	1.09
C18:2, %	1.95	1.90	1.80	1.40	1.40	1.45
C18:3, %	0.10	0.16	0.45	0.07	0.12	0.36
SFA, %	0.55	0.54	0.55	1.01	0.98	1.00
USFA, %	1.10	1.08	1.26	0.80	0.82	0.96
LA:ALA	19.50	11.88	4.00	20.00	11.67	4.03
U:S	2.00	2.00	2.29	0.79	0.84	0.96
Phase 2						
C12:0, %	–	–	–	–	–	0.14
C14:0, %	–	–	–	0.03	0.03	0.06
C18:0, %	0.07	0.07	0.09	0.30	0.29	0.28
C18:1, %	1.11	1.10	1.07	1.27	1.25	1.04
C18:2, %	2.00	1.96	1.80	1.41	1.41	1.42
C18:3, %	0.10	0.17	0.45	0.07	0.12	0.36
SFA, %	0.57	0.56	0.55	1.05	1.02	0.98
USFA, %	1.11	1.12	1.19	0.75	0.76	0.77
LA:ALA	20.00	11.53	4.00	20.14	11.75	3.94
U:S	1.95	2.00	2.16	0.71	0.75	0.79
Phase 3						
C12:0, %	–	–	–	–	–	0.15
C14:0, %	–	–	–	0.03	0.02	0.06
C18:0, %	0.07	0.07	0.09	0.27	0.27	0.26
C18:1, %	1.12	1.11	1.08	1.27	1.25	1.03
C18:2, %	2.00	1.96	1.80	1.47	1.47	1.47
C18:3, %	0.10	0.16	0.45	0.07	0.12	0.37
SFA, %	0.57	0.57	0.56	1.00	0.97	0.95
USFA, %	1.05	1.05	1.12	0.72	0.74	0.74
LA:ALA	20.00	12.25	4.00	21.00	12.25	3.97
U:S	1.84	1.84	2.00	0.72	0.76	0.78

^1^Dietary FA ratio as dietary LA:ALA (LA:ALA): 20:1, 12:1, and 4:1.

SFA, saturated fatty acids; USFA, unsaturated fatty acids.

### Sample Collection

Pigs were individually weighed and feed disappearance was recorded on days 0, 14, 42, and 70 to calculate ADG, average daily feed intake (ADFI), and gain to feed ratio (G:F) for pen within each phase. On day 49, one vaccinated gilt per pen was moved to individual housing (pen size = 1.41 m × 2.18 m) at approximately 154 d of age for evaluation of reproductive characteristics. One vaccinated barrow per pen was also removed to maintain equal pig space across all pens. Gilts remained on their experimental diets for the remainder of the study. Gilts were administered 4 mL of boar saliva pheromones (BOAR BETTER, Dallas, TX) intranasally twice a day at 0630 and 1630 hours. Gilts were evaluated by trained researchers for signs of estrus, including standing heat, erect ears, swollen and red vulvas, and vaginal discharge. Upon detection of previously described signs of estrus, gilts stopped receiving boar pheromones.

Eighteen days postestrus detection, gilts were administered 4 mL of boar saliva pheromones intranasally twice a day at 0630 and 1630 hours and observed for signs of estrus. Upon detection of their second estrus, gilts were euthanized by captive-bolt followed by exsanguination and the right ovaries were collected. Gilts that did not display signs of estrus by day 46 postplacement into individual housing were euthanized by captive-bolt followed by exsanguination and the right ovary was collected for evaluation of ovarian structure development to determine if gilts had cycled.

### Chemical Analysis

Diets were ground to 1 mm particle size with a Wiley Mill (Variable Speed Digital ED-5 Wiley Mill; Thomas Scientific, Swedesboro, NJ) and analyzed in duplicate for dry matter (method 930.15 [AOAC, 2007]), acid-hydrolyzed ether extract (aEE; method 2003.06; [AOAC, 2007]), and N (method 990.03 [AOAC, 2007]; TruMac; LECO Corp., St. Joseph, MI). An EDTA sample (9.56% N) was used as the standard for calibration and was determined to contain 9.55% ± 0.01% N. Crude protein was calculated as N × 6.25. Gross energy (GE) was determined in duplicate using an isoperibolic bomb calorimeter (model 6200; Parr Instrument Co., Moline, IL). Benzoic acid (6,318 kcal GE/kg) was used as the standard for calibration and was determined to contain 6,319 ± 0.8 kcal GE/kg.

Total lipids were extracted by using a chloroform and methanol mixture (Folch et al., 1957). The lipids were methylated directly with acetyl chloride and methanol (Christie et al., 1972). FA methyl esters were quantified by a gas chromatograph (Varian 3800, Agilent Technologies, Palo Alto, CA) equipped with a Supelco Sp-2380 column and a flame ionization detector. Gas chromatograph conditions were as follows: initial column temp, 70 °C with a hold time of 4 min, temperature ramp was 13 °C per minute with a final column temperature of 215 °C. Peaks were identified by using commercially available FA methyl ester standards (Nu-Chek-Prep Inc., Elysian, MN).

### Statistical Analysis

Growth performance data by phase were analyzed as repeated measures using the MIXED procedure of SAS 9.4 (SAS Inst.). Pen was the experimental unit, with U:S, LA:ALA, sex, phase, and their interactions included as fixed effects in the statistical model. Initial BW was fit as a covariate. The spatial power covariance structure was selected for the repeated measures model according to Bayesian information criterion. Statistical outliers were identified as occurring greater than three standard deviations from the mean of the studentized residuals and were excluded from the analysis. Normality and homoscedasticity of the studentized residuals were tested using the UNIVARIATE procedure. Data were reported as least-squares means and means separation was done using the PDIFF option. Gilt estrus detection data were analyzed using the GENMOD procedure of SAS 9.4 with a binary distribution and a logit link function. Data were reported as raw frequencies and percentages with the resulting *P*-value from the model. Results were considered significant at *P* ≤ 0.05 and a trend at *P* > 0.05 and *P* ≤ 0.10.

## Results and Discussion

Previous research indicates that feeding growing pigs differing in dietary LA:ALA can alter growth performance of gilts (Becker and Greiner, 2021; Becker et al., 2023). The current study aimed to investigate if these same previously researched dietary LA:ALA ratios impacted growth performance of growing pigs and estrus detection of gilts when formulated with differing dietary U:S ratios. Within a phase, there were no differences in BW (*P* ≥ 0.461), ADG (*P* ≥ 0.128), ADFI (*P* ≥ 0.358), or feed efficiency (*P* ≥ 0.461) for the effects of U:S, LA:ALA, or their interactions (Table 6). The U:S, LA:ALA, or their interaction did not impact overall (days 0 to 70) growth performance (*P* ≥ 0.128; Table 7). Similar results have been shown in a previous study by Woodworth et al. (1999) evaluating differing dietary fat sources on growth performance of grow-finish pigs. Stephenson et al. (2016) fed three dietary fat sources, either soybean oil, tallow, or a blend of soybean oil and tallow, to finishing pigs for three different feeding durations (days 0 to 42, 42 to 84, or 0 to 84). During the first period, pigs fed diets with added soybean oil or tallow had improved feed efficiency compared to pigs fed diets containing the blend of soybean oil and tallow, with no differences of fat sources during the second period. Overall, pigs fed soybean oil, a highly unsaturated fat, tended to have improved feed efficiency compared to those receiving diets formulated with the blend of soybean oil and tallow, though not different from tallow, regardless of sex. There were no differences between fat sources for ADG, ADFI, or BW at any feeding duration (Stephenson et al., 2016). In contrast, previous literature has shown that growing pigs fed soybean oil, a highly unsaturated fat source, results in improved ADG compared to growing pigs fed beef tallow, a highly saturated fat source (Morgan et al., 1992). There was an effect of the U:S × LA:ALA interaction for overall ADG. Pigs receiving the HUS × 20:1 and LUS × 12:1 diets had improved ADG over pigs receiving the HUS × 12:1 diet (*P *= 0.043; Table 7).

**Table 6.** The simple effects of U:S and essential FA ratio on growth performance and feed efficiency of grow-finish pigs by weigh period^1^

	U:S ratio^2^		FA ratio^3^			SEM	*P*-value^4^		
Item	High	Low	20:1	12:1	4:1		Period	U:S × Period	FA × Period
BW, kg						1.206	< 0.001	0.467	0.461
Day 0	54.39	54.40	54.39	54.39	54.41				
Day 14	69.79	69.80	70.00	69.42	69.97				
Day 42	102.08	103.20	102.47	103.42	102.03				
Day 70	133.84	134.33	134.20	134.59	133.46				
ADG, kg						0.031	< 0.001	0.333	0.607
Days 0–14	1.08	1.10	1.09	1.08	1.11				
Days 14–42	1.19	1.21	1.19	1.22	1.19				
Days 42–70	1.13	1.11	1.13	1.11	1.12				
ADFI, kg						0.058	< 0.001	0.495	0.448
Days 0–14	2.46	2.44	2.47	2.42	2.46				
Days 14–42	3.04	3.07	3.02	3.08	3.06				
Days 42–70	3.18	3.23	3.20	3.20	3.21				
G:F						0.010	< 0.001	0.467	0.461
Days 0–14	0.44	0.45	0.45	0.44	0.44				
Days 14–42	0.39	0.40	0.39	0.40	0.40				
Days 42–70	0.36	0.34	0.35	0.35	0.35				

^1^Data are least square means; *N* = 10 pens per treatment with 4 pigs per pen, totaling 240 pigs; growth calculations included pig days to account for morbidity and mortality.

^2^Calculated dietary U:S - High = >1.8, Low = <1.0.

^3^Calculated FA ratio as dietary LA:ALA.

^4^Across all variables, there were no differences for the Period × U:S × FA interaction.

**Table 7.** The main effects of U:S and essential FA ratio on overall growth performance and feed efficiency of grow-finish pigs^1^

	U:S^2^ > 1.8			U:S < 1.0			SEM	*P*-value^3^		
Item	20:1^4^	12:1	4:1	20:1	12:1	4:1		U:S	FA	U:S × FA
ADG, kg	1.17^a^	1.12^b^	1.15^a,b^	1.16^a,b^	1.19^a^	1.15^a,b^	0.017	0.186	0.604	0.043
ADFI, kg	2.97	2.91	2.98	2.93	3.07	3.00	0.049	0.189	0.595	0.114
G:F	0.40	0.39	0.39	0.39	0.39	0.38	0.005	0.385	0.433	0.472

^1^Data are least square means; *N* = 10 pens per treatment with 4 pigs per pen, totaling 240 pigs; growth calculations included pig days to account for morbidity and mortality.

^2^Calculated dietary U:S - High = >1.8, Low = <1.0.

^3^Within a dependent variable, means without a common superscript differ significantly (*P < *0.05).

^4^Calculated FA ratio as dietary LA:ALA.

Barrows had higher final BW, ADG, and ADFI across each phase (*P ≤ *0.035; Table 7) and for the overall days 0 to 74 period (*P < *0.001; Table 9) compared to gilts. There was a period × sex interaction for feed efficiency (*P < *0.001; Table 8). Gilts had a higher feed efficiency from days 14 to 42; however, feed efficiency was not different between barrows and gilts from days 0 to 14 and 42 to 70; resulting in a similar feed efficiency between sexes for the overall period (*P* = 0.523; Table 9). These growth differences between sexes agree with previously reported data (Faccin et al., 2022). Compared to LUS, gilts receiving HUS had lower ADFI overall (*P = *0.018), which translated into improved G:F for HUS gilts (*P = *0.011). Limited data exist evaluating the interactions of dietary fat source and sex on pig growth performance. Tartrakoon et al. (2016) observed an improvement in the feed efficiency of gilts fed a U:S of 5:1 compared to 2.5:1 in diets formulated with combination of coconut and canola oils. However, Benz et al. (2011) did not observe an interaction between sex and dietary fat source as either choice white grease (~7:1 LA:ALA) or soybean oil (~1:1 LA:ALA) on growth performance of grow-finish pigs, though soybean oil improved ADG overall.

**Table 8.** The simple effects of U:S, essential FA ratio, and sex on growth performance and feed efficiency of grow-finish pigs by weigh period^1^

	U:S ratio^2^				FA ratio^3^						SEM	*P*-value^4^		
Item	High		Low		20:1		12:1		4:1			Period × Sex	Period × Sex × U:S	Period × Sex × FA
	M	F	M	F	M	F	M	F	M	F				
BW, kg											1.708	< 0.001	0.696	0.769
Day 0	54.43	54.36	54.39	54.40	54.40	54.37	54.41	54.37	54.42	54.39				
Day 14	71.00	68.58	70.93	68.68	71.04	68.96	70.80	68.05	71.06	68.88				
Day 42	104.34	99.82	106.20	100.20	104.63	100.31	106.73	100.12	104.46	99.61				
Day 70	138.29	129.38	139.77	128.89	138.03	130.37	140.69	128.50	138.38	128.54				
ADG, kg											0.044	0.035	0.673	0.785
Days 0–14	1.18	0.99	1.18	1.02	1.19	0.99	1.17	0.98	1.18	1.04				
Days 14–42	1.23	1.15	1.27	1.16	1.24	1.15	1.27	1.16	1.24	1.15				
Days 42–70	1.21	1.06	1.20	1.02	1.19	1.08	1.21	1.02	1.21	1.03				
ADFI, kg											0.083	< 0.001	0.450	0.597
Days 0–14	2.66	2.26	2.60	2.29	2.67	2.27	2.56	2.29	2.66	2.27				
Days 14–42	3.30	2.78	3.30	2.84	3.28	2.77	3.30	2.85	3.32	2.81				
Days 42–70	3.34	3.03	3.40	3.05	3.33	3.07	3.36	3.04	3.41	3.01				
G:F											0.015	< 0.001	0.908	0.855
Days 0–14	0.44	0.44	0.46	0.45	0.45	0.44	0.46	0.43	0.45	0.46				
Days 14–42	0.37	0.41	0.38	0.41	0.38	0.42	0.39	0.41	0.37	0.41				
Days 42–70	0.36	0.35	0.35	0.34	0.36	0.35	0.36	0.33	0.36	0.34				

^1^Data are least square means; *N* = 10 pens per treatment with 4 pigs per pen, totaling 240 pigs; growth calculations included pig days to account for morbidity and mortality.

^2^Calculated dietary U:S - High = >1.8, Low = <1.0.

^3^Calculated FA ratio as dietary LA:ALA.

^4^For all variables, there were no differences for the interaction of Period × U:S × FA, Sex × U:S × FA, or Period × Sex × U:S × FA (*P* > 0.10).

M, male; F, female.

**Table 9.** The main effects of U:S, essential FA ratio, and sex on overall growth performance and feed efficiency of grow-finish pigs^1^

	U:S ratio^2^				FA ratio^3^						SEM	*P*-value^4^		
Item	High		Low		20:1		12:1		4:1			Sex	Sex × U:S	Sex × FA
	M	F	M	F	M	F	M	F	M	F				
ADG, kg	1.22	1.08	1.22	1.11	1.21	1.12	1.23	1.09	1.22	1.08	0.026	< 0.001	0.370	0.205
ADFI, kg	3.21^a^	2.70^c^	3.17^a^	2.84^b^	3.17	2.73	3.16	2.82	3.23	2.74	0.073	< 0.001	0.018	0.291
G:F	0.38^b^	0.40^a^	0.39^b^	0.39^b^	0.38	0.40	0.39	0.39	0.38	0.39	0.007	0.526	0.011	0.086

^1^Data are least square means; *N* = 10 pens per treatment with 4 pigs per pen, totaling 240 pigs; growth calculations included pig days to account for morbidity and mortality.

^2^Calculated dietary U:S - High = >1.8, Low = <1.0.

^3^Calculated FA ratio as dietary LA:ALA.

^4^Within a dependent variable, means without a common superscript differ significantly (*P < *0.05). There were no differences for the interaction of Sex × U:S × FA (*P *> 0.10).

M, male; F, female.

Additionally, gilts receiving the 20:1 diet in the current study tended to have improved G:F compared to 12:1 for the days 0 to 70 period (*P *= 0.086; Table 9). An improvement in overall ADG when feeding a 20:1 FA ratio was also observed by Becker and Greiner (2021) in growing gilts. There was no difference in gilt estrus detection for U:S (*P = *0.737; Table 10), FA (*P = *0.361) or U:S × FA (*P = *0.356). Minimal research has been completed investigating the optimum dietary FA profile for the developing gilt prior to puberty onset. Vaccinations were given prior to puberty to simulate commercial production practices in the gilt developer unit. Given the limited number of animals utilized in this subset and low detection of estrus overall, further research is needed to investigate the impact of dietary essential FAs on reproductive characteristics of developing gilts. Additionally, heat detection in this study occurred during the months of June and July in central Iowa. It is possible heat stress associated with high ambient temperatures during this time delayed the onset of puberty (Flowers et al., 1989). Photoperiod can also play a role in puberty attainment of gilts. The average day length during the current study was approximately 15 h, and previous research has demonstrated that when gilts experience of day length of over 12 h, natural onset of puberty is delayed (Paterson and Pearce, 1990; Patterson et al., 1991). It is hypothesized that these changes in gilt growth performance could be a result of altering hormone production, which are end products of FA and cholesterol metabolism, and delaying the onset of estrus (Bhathena, 2000). Chartrand et al. (2003) reported that dietary supplementation of flaxseed oil reduced prostaglandin in postpubertal gilts, both pre- and postmating. This hypothesis is also supported by the low detection rate of estrus in the subset of gilts in the current study, though further research is warranted to validate this hypothesis. LA and ALA are also precursors for inflammatory metabolites. Previous research by Duan et al. (2014) reported that a dietary LA:ALA of 1:1 to 5:1 was optimal for reducing inflammatory markers in tissue and circulation, with pigs fed a 5:1 LA:ALA having improved growth performance. This contrasts studies by Becker et al. (2023) and Becker and Greiner (2021) where no changes in inflammatory metabolites in grow-finish pigs fed differing LA:ALA ratios were observed. Inhibition of inflammation through a reduction in dietary LA:ALA could allow more energy availability for improved performance; however, further investigation into this relationship is required in pigs.

**Table 10.** The main effects of U:S and essential FA ratio on the detection of the first-estrus of gilts by 235 d of age

	U:S^1^ > 1.8			U:S < 1.0			*P*-value		
	20:1^2^	12:1	4:1	20:1	12:1	4:1	U:S	Ratio	U:S × Ratio
Percent							0.737	0.361	0.356
Yes	60%	60%	20%	20%	60%	40%			
No	40%	40%	80%	60%	40%	60%			

^1^Calculated dietary U:S - High = >1.8, Low = <1.0.

^2^Calculated FA ratio as dietary LA:ALA.

In conclusion, the data presented herein demonstrates an improvement in feed efficiency of growing gilts when fed diets formulated with unsaturated fat sources. This improvement in feed efficiency was also observed in gilts fed a 20:1 essential FA ratio, independent of fat source. When feeding a diet containing unsaturated fats, a 20:1 LA:ALA improves gain over 12:1 essential FA ratio; however, a lower LA:ALA is optimal when feeding diets high in saturated fats. ADG was optimized in pigs fed diets formulated with unsaturated fat sources to a 20:1 LA:ALA, regardless of sex. Additional investigation into the biological mechanisms impacting the growth of gilts fed varying dietary essential FA ratios is needed to understand the potential positive or negative consequences on reproductive outcomes.

**Table 2.** Ingredient and nutrient composition of grow-finish experimental diets, phase 2 (as-fed basis)

Item	U:S^1^ > 1.8			U:S > 1.0		
	20:1^2^	12:1	4:1	20:1	12:1	4:1
Ingredient, %						
Corn	77.53	77.52	77.43	77.39	77.40	77.39
Soybean meal	18.13	18.13	18.13	18.13	18.13	18.13
Beef tallow	–	–	–	0.73	0.78	0.52
Choice white grease	–	–	–	0.68	0.53	–
Corn oil	1.21	1.1	0.64	–	–	–
Flaxseed oil	0.07	0.19	0.74	0.02	0.12	0.57
Palm kernel oil	–	–	–	–	–	0.35
DL-methionine	0.06	0.06	0.06	0.06	0.06	0.06
L-lysine HCL	0.34	0.34	0.34	0.34	0.34	0.34
L-threonine	0.11	0.11	0.11	0.11	0.11	0.11
L-tryptophan	0.01	0.01	0.01	0.01	0.01	0.01
Premix^3^	0.35	0.35	0.35	0.35	0.35	0.35
Phytase^4^	0.02	0.02	0.02	0.02	0.02	0.02
Calcium carbonate	1.06	1.06	1.06	1.07	1.07	1.07
Monocalcium phosphate	0.62	0.62	0.62	0.60	0.60	0.60
Sodium chloride	0.50	0.50	0.50	0.50	0.50	0.50
Calculated nutrient levels						
SID Lys, %	0.89	0.89	0.89	0.88	0.88	0.88
SID Met: Lys	0.32	0.32	0.32	0.32	0.32	0.32
SID Thr: Lys	0.63	0.63	0.63	0.63	0.63	0.63
SID Trp:Lys	0.18	0.18	0.18	0.18	0.18	0.18
SID TSAA: Lys	0.57	0.57	0.57	0.57	0.57	0.57
Ca, %	0.70	0.70	0.70	0.70	0.70	0.70
STTD P, %	0.35	0.35	0.35	0.35	0.35	0.35
ME, Mcal/kg	3.34	3.34	3.35	3.34	3.34	3.34
SID Lys/ME, (g/kg)/Mcal	2.65	2.65	2.65	2.65	2.65	2.65
Analyzed nutrient levels						
Dry matter, %	87.67	87.73	88.34	88.21	88.38	87.79
GE, Mcal/kg	3.85	3.87	3.89	3.89	3.87	3.89
aEE, %	3.99	4.31	3.53	4.26	4.61	4.80

^1^Calculated dietary unsaturated:saturated fat ratio (U:S): High = >1.8, Low = <1.0.

^2^Calculated FA ratio as dietary LA:ALA.

^3^Provided 6,614 IU vitamin A, 827 IU vitamin D, 26 IU vitamin E, 2.6 mg vitamin K, 29.8 mg niacin, 16.5 mg pantothenic acid, 5.0 mg riboflavin, and 0.023 mg vitamin B12, 165 mg Zn (zinc sulfate), 165 mg Fe (iron sulfate), 39 mg Mn (manganese sulfate), 17 mg Cu (copper sulfate), 0.3 mg I (calcium iodate), and 0.3 mg Se (sodium selenite) per kg of diet.

^4^450 FTU/kg of diet provided 0.06% Ca and 0.11% STTD P.

ME, metabolizable energy; STTD, standardized total tract digestible; SID, standardized ileal digestible; aEE, acid ether extract.

**Table 3.** Ingredient and nutrient composition of grow-finish experimental diets, phase 3 (as-fed basis)

Item	U:S^1^ > 1.8			U:S < 1.0		
	20:1^2^	12:1	4:1	20:1	12:1	4:1
Ingredient, %						
Corn	82.35	82.38	82.25	82.25	82.26	82.24
Soybean meal	13.31	13.31	13.31	13.31	13.31	13.31
Beef tallow	–	–	–	0.54	0.58	0.40
Choice white grease	–	–	–	0.77	0.63	–
Corn oil	1.14	1.02	0.56	–	–	–
Flaxseed oil	0.08	0.19	0.74	0.04	0.13	0.60
Palm kernel oil	–	–	–	–	–	0.36
DL-methionine	0.03	0.03	0.03	0.03	0.03	0.03
L-lysine HCL	0.34	0.34	0.34	0.34	0.34	0.34
L-threonine	0.10	0.10	0.10	0.10	0.10	0.10
L-tryptophan	0.02	0.02	0.02	0.02	0.02	0.02
Premix^3^	0.35	0.35	0.35	0.35	0.35	0.35
Phytase^4^	0.02	0.02	0.02	0.02	0.02	0.02
Calcium carbonate	1.06	1.08	1.05	1.08	1.08	1.08
Monocalcium phosphate	0.72	0.66	0.74	0.66	0.66	0.66
Sodium chloride	0.50	0.50	0.50	0.50	0.50	0.50
Calculated nutrient levels^5^						
SID Lys, %	0.77	0.77	0.77	0.77	0.77	0.77
SID Met: Lys	0.31	0.31	0.31	0.31	0.31	0.31
SID Thr: Lys	0.63	0.63	0.63	0.63	0.63	0.63
SID Trp:Lys	0.18	0.18	0.18	0.18	0.18	0.18
SID TSAA: Lys	0.57	0.57	0.57	0.57	0.57	0.57
Ca, %	0.70	0.70	0.70	0.70	0.70	0.70
STTD P, %	0.36	0.35	0.36	0.35	0.35	0.35
ME, Mcal/kg	3.34	3.34	3.34	3.34	3.34	3.34
SID Lys/ME, (g/kg)/Mcal	2.30	2.30	2.30	2.30	2.30	2.30
Analyzed nutrient levels^6^						
Dry matter, %	87.85	88.02	88.39	88.25	88.08	87.84
GE, Mcal/kg	3.83	3.83	3.85	3.84	3.87	3.86
aEE, %	4.54	4.54	4.34	4.68	4.26	4.65

^1^Calculated dietary U:S - High = >1.8, Low = <1.0.

^2^Calculated FA ratio as dietary LA:ALA.

^3^Provided 6,614 IU vitamin A, 827 IU vitamin D, 26 IU vitamin E, 2.6 mg vitamin K, 29.8 mg niacin, 16.5 mg pantothenic acid, 5.0 mg riboflavin, and 0.023 mg vitamin B12, 165 mg Zn (zinc sulfate), 165 mg Fe (iron sulfate), 39 mg Mn (manganese sulfate), 17 mg Cu (copper sulfate), 0.3 mg I (calcium iodate), and 0.3 mg Se (sodium selenite) per kg of diet.

^4^450 FTU/kg of diet provided 0.06% Ca and 0.11% STTD P.

ME, metabolizable energy; STTD, standardized total tract digestible; SID, standardized ileal digestible; aEE, acid ether extract.
